# Distribution of nitric oxide-producing cells along spinal cord in urodeles

**DOI:** 10.3389/fncel.2014.00299

**Published:** 2014-09-25

**Authors:** Mayada A. Mahmoud, Gehan H. Fahmy, Marie Z. Moftah, Ismail Sabry

**Affiliations:** ^1^Faculty of Medicine, Institut de Neurosciences des Systèmes, Unités Mixtes de Recherche Institut National de la Santé et de la Recherche Médicale 1106, Aix-Marseille UniversityMarseille, France; ^2^Zoology Department, Faculty of Science, Alexandria UniversityAlexandria, Egypt

**Keywords:** nitric oxide, spinal cord, urodeles, NADPH-d labeling, neurotransmitter agents

## Abstract

Nitric oxide is a unique neurotransmitter, which participates in many physiological and pathological processes in the organism. There are little data about the neuronal nitric oxide synthase immunoreactivity in the spinal cord of amphibians. In this respect, the present study aims to investigate the distribution of nitric oxide producing cells in the spinal cord of urodele and to find out the possibility of a functional locomotory role to this neurotransmitter. The results of the present study demonstrate a specific pattern of NADPH-d labeling in the selected amphibian model throughout the spinal cord length as NADPH-d-producing cells and fibers were present in almost all segments of the spinal cord of the salamander investigated. However, their number, cytological characteristics and labeling intensity varied significantly. It was noticed that the NO-producing cells (NO-PC) were accumulated in the ventral side of certain segments in the spinal cord corresponding to the brachial and sacral plexuses. In addition, the number of NO-PC was found to be increased also at the beginning of the tail and this could be due to the fact that salamanders are tetrapods having bimodal locomotion, namely swimming and walking.

## Introduction

Nitric oxide (NO) was recognized as the first gaseous neurotransmitter with a very short half-life time (2–6 s) (Greenwood and Earnshaw, 1997; Barañano et al., 2001; González-Soriano et al., 2002) and it has been implicated as a non-adrenergic non-cholinergic (NANC) inhibitory neurotransmitter at various sites in the nervous system (Grozdanovic et al., 1994; Schuman and Madison, 1994; Sharma et al., 2005).

Anatomical studies have linked NO to developing brain regions associated with locomotion in the rat (Terada et al., 1996), fish (Villani, 1999) and even insects (Wildemann and Bicker, 1999). In addition, it is widely accepted that NO plays a major role in sensory and motor systems (Funakoshi et al., 1999), neurogenesis (Estrada and Murillo-Carretero, 2005) and neuroendocrine and autonomic nervous activities (Gerstberger, 1999; Guo and Longhurst, 2003). There is vast evidence that NO is also involved in spinal functions. Its effects include the regulation of cardiovascular function and the mediation of nociception (Chowdhary and Townend, 1999; Osuka et al., 2008), and it is well accepted that NO is involved in nociceptive processing and persistent pain as intracellular and intercellular messengers in the spinal cord (Ito et al., 2001; Chung et al., 2005; Dagci et al., 2011).

NO is a typical neurotransmitter which is a typical in chemical nature, biosynthesis, mechanism of action, and cellular localization as they are neither stored in synaptic vesicles nor released by exocytosis. They nearly diffuse into adjacent neurons (Barañano et al., 2001) where they block cellular enzymes required in metabolism and activate soluble guanylate cyclase (sGC)—an insoluble enzyme—in an inactive form, present in the cells cytoplasm. The activation occurs via interaction of NO with Fe^+2^ in the heme portion of the molecule, thereby altering its conformation and activating it, this causes conversion of guanosine triphosphate (GTP) to cyclic guanosine monophosphate (cGMP), which mediates many of the physiological actions of NO in mammalian cells (McCann et al., 2005). In aldehyde fixed tissue, nitric oxide synthase (NOS) is able to selectively catalyze a histochemically detectable Golgi-like dense staining of reactive neurons produced by Nicotinamide Adinine Dinulceotide Phosphate diaphorase (NADPH-d) histochemistry (González-Soriano et al., 2002). This is known as NADPH-diaphorase activity, which is used as a histochemical detection method for neuronal NO-producing structures (Bredt et al., 1991).

Nitrergic elements have been inferred from cells positive to NADPH-d histochemistry and/or to the neuronal nitric oxide synthase (nNOS) immunohistochemistry in different species of vertebrates (Giraldez-Perez et al., 2008). Thus, the distribution of neuronal elements that express NOS in the brain of the amphibian *Dermophis mexicanus*, by means of immunohistochemistry, with specific antibodies against NOS, and enzyme histochemistry for NADPH-diaphorase were equally demonstrated by both techniques (González et al., 2002).

The most important and attractive reason, for which neuroanatomists were interested in the technique of detecting NADPH-d by histochemistry, arose when NADPH was identified as a marker for nNOS (Hope et al., 1991). It has been repeatedly corroborated that in the nervous system, NADPH activity and NOS immunoreactivity widely colocalize in distinct sets of neurons (Briñon et al., 1998; Giraldez-Perez et al., 2008). The relative simple NADPH-d histochemical technique was widely used to identify NO-producing elements in the brain of representatives of all vertebrate classes (Arévalo et al., 1995; Munoz et al., 1996; Smeets et al., 1997; Alonso et al., 2000).

While several studies have described the localization of NO-PC in the developing brains of birds and mammals including human brain (Vincent, 1994; Samama et al., 1995; Takemura et al., 1996; Terada et al., 1996; Iwase et al., 1998), the distribution of NO-positive cell population has not been well characterized in the amphibian central nervous system. In fact, the spinal cord (Thomas and Pearse, 1964)—which has extensive distribution of NOS-containing cells and fibers—and the brain of amphibians have been demonstrated for several species of anurans (Lázár and Losonczy, 1999; López and González, 2002) and urodeles (González et al., 1996; Moreno et al., 2002). Salamanders (a urodele amphibian animal) is a perfect animal model for studying the relation between spinal cord and movements, as they do not lose their tails after metamorphosis and thereby conceives the ability for both axial and limb-based locomotion in adulthood.

There is little data about the neuronal nitric oxide synthase immunoreactive (nNOS-ir) neurons of amphibian spinal cord. In this respect, the aims of this study were to demonstrate the presence of NO in neurons of salamanders' spinal cord, to describe its light microscope morphology and distribution, to verify whether the NO-producing neurons have specific patterns of organization throughout the spinal cord and to investigate whether this transmitter has a certain relation with movement or not.

## Materials and methods

### Histological preparation

A group of four salamanders were anesthetized by immersion in a 0.1% aqueous solution Tricaine methane sulphonate (MS-222; Sigma, Saint Quentin Fallavier, France). Vertebral columns were separated and fixed in Bouin's solution for two h at room temperature. The spinal cord was dissected out by cutting the vertebral column after each two constitutive vertebrae starting from the Atlas except vertebra number 9 and withdrawing it anteriorly. It has been thus divided anatomically into five segments. As for segment V, it represents the part of the spinal cord present within vertebra number 9 (sacral).

Collected spinal cord specimens were dehydrated in an ascending series of ethanol (60 m for each), cleared twice in xylene (60 m each), impregnated in wax and xylene for 10 m (1:1), embedded twice in paraffin wax (1 h. each), then finally embedded in paraffin wax. Specimens were then sectioned (5 μm thick) by using a traditional mictrotome (American Optical Scientific Instrument Division, Buffalo, NY, USA), then stained with hematoxylin and eosin for microscopic histological study. Cell profiles were confirmed by adjusting the focal depth of the objective.

### NADPH-d histochemistry

Salamanders were perfused transcardially with oxygenated urodele Ringer's solution as previously tested (Chevallier et al., 2004) and then by 4% paraformaldehyde in 0.1 M phosphate buffer (pH 7.4). The dorsal half of the vertebrae was removed (laminectomy). The spinal cord was separated from the opened vertebrae and divided into five segments for histological preparations. Specimens were post-fixed in 4% paraformaldehyde for 2 h at room temperature then immersed in a solution of 12% sucrose in PB overnight at 4°C. Specimens were transferred into tissue Tek and kept in −80°C until processed. Blocks were transferred to −20°C for 2 h before being cut on a cryostat at 15 μm. Sections were collected in phosphate buffer (PB) as free-floating sections. They were rinsed in fresh PB then treated according to previously published method (Moreno et al., 2002). In brief, free-floating sections were incubated in a medium containing 1 mM β-NADPH, 0.8 mM nitro blue tetrazolium and 0.06% triton X-100 in PB, at 37°C for 1–2 h. The reaction was stopped by successive rinses in cold PB. Some sections were incubated in a medium without β-NADPH to be used as controls. All sections were then mounted using 0.25% gelatin in 0.1 M Tris buffer, pH 7.6 then dried overnight and cover slipped.

### Image acquisition

The distribution of nitric oxide positive cells (NO-PC) in the spinal cord was charted in a series of transverse sections along the rostrocaudal axis of the animal by using camera Lucida and the image of each segment was recorded with a digital camera wide zoom operating on a microscope, and reversed on a computer by the Analysis Life Science Series (Soft Imaging System, Japan) and Life View software (Animation Technologies Inc, Taiwan).

### Quantification

Quantification procedures have been done by drawing all sections using camera lucida, then manually counting all stained cells. All neurons that contained violet stain (strong stain = dark violet color, weak stain = light violet color) in all sections incubated in NADPH-d were expressed as mean ± SE.

### Statistical analysis

All data were represented as means ± s.e.m. and were statistically analyzed with One-Way ANOVA by using “Statistical Package for the Social Sciences” SPSS software and student's two-tailed *t*-test to compare each two groups together.

## Results

**Figure 1:**
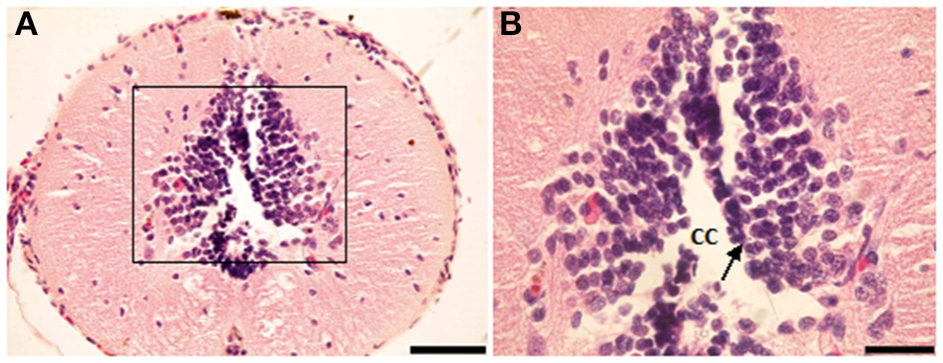
**Photomicrograph showing transverse sections through the spinal cord segment IA**. **(B)** is enlargement of the framed area in **(A)**, note the shape of ependymal cells (arrow). CC, central canal. Scale bar in **(A)** is 100 μm and in **(B)** is 50 μm.

In the salamander's spinal cord, the gray matter was composed of unspecialized primitive cells (Figures 1A,B). They were arranged in 9–10 concentric rows surrounding the central canal. Although not specialized, the first internal raw of cells lining the central canal was oriented toward its cavity, hence referred to as ependymal cells.

**Figure 2:**
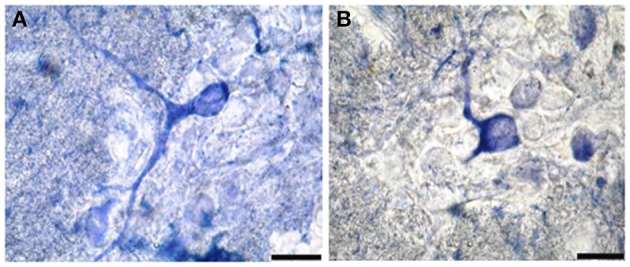
**Photomicrograph showing different shapes of NO-PC in the spinal cord of salamanders**. **(A)** Unipolar neuron. Note the axon extending from the cell body toward the white matter. **(B)** Bipolar neuron. Scale bar: 20 μm.

NO-PC and fibers were found to be present in almost all segments of the spinal cord. They could also be divided into two classes: (1) heavily stained indicating large quantity of NO production and (2) lightly stained neurons indicating less quantity of NO production. For the latter, only cell bodies were observed, whereas for the former, cell bodies and their dendrites were both visible. NADPH-d labeling clearly revealed discrete populations of pear-shaped cells, which may be unipolar and/or bipolar neurons (Figures 2A,B).

**Figure 3:**
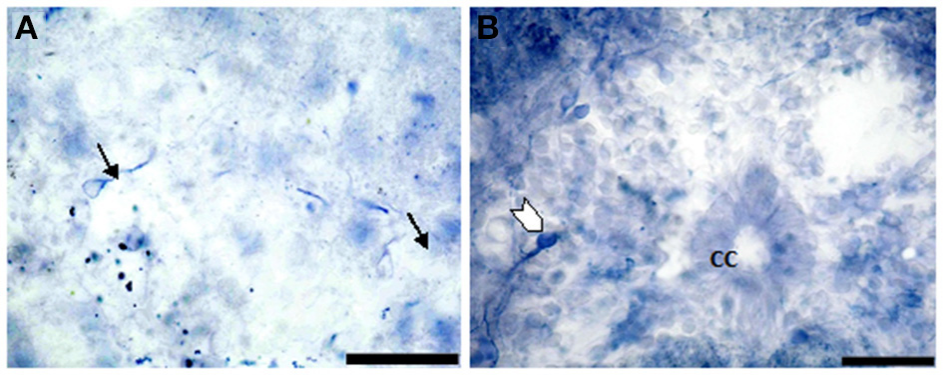
**Photomicrograph showing the distribution of NO-PC in segment IA (arrows in A) and IP (arrowhead in B) in salamanders**. Note the difference in stained neurons (weak: arrows in **A** and strong: arrowhead in **B**). Scale bar is 50 μm.

**Figure 4:**
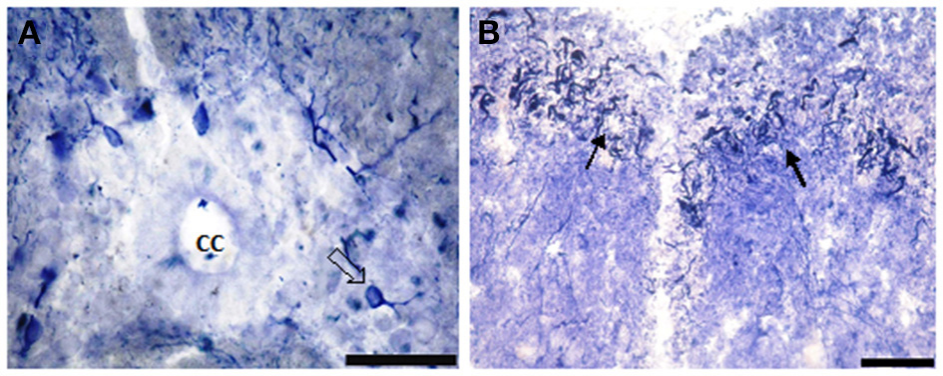
**Photomicrograph showing NO-PC distribution in spinal cord segment II of salamanders**. Note the presence of the densely-packed multipolar neurons and moderate staining pear-shaped neurons (**B**: arrows). Scale bar in **(A)** is 50 μm and in **(B)** is 100 μm.

**Figure 5:**
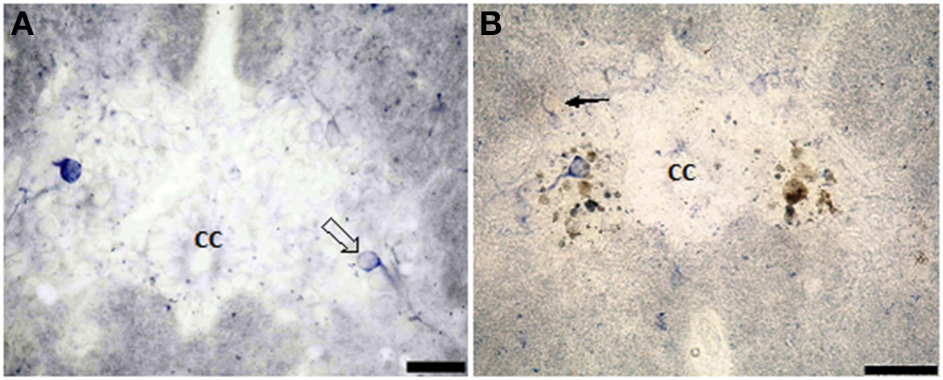
**Photomicrograph showing the distribution of NO-PC in segment III (A) and segment IV (B) in salamanders**. Scale bar is 50 μm.

**Figure 6:**
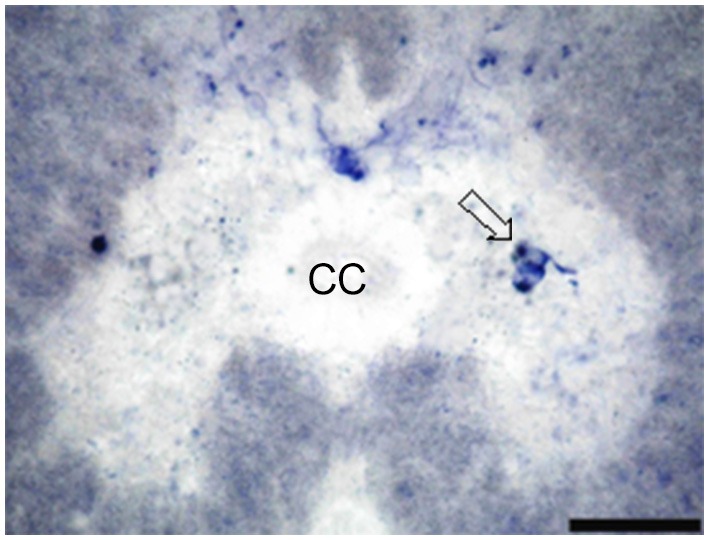
**Photomicrograph showing the distribution of NO-PC in the last studied segment of salamanders spinal cord, namely segment V**. Scale bar is 50 μm.

**Figure 7:**
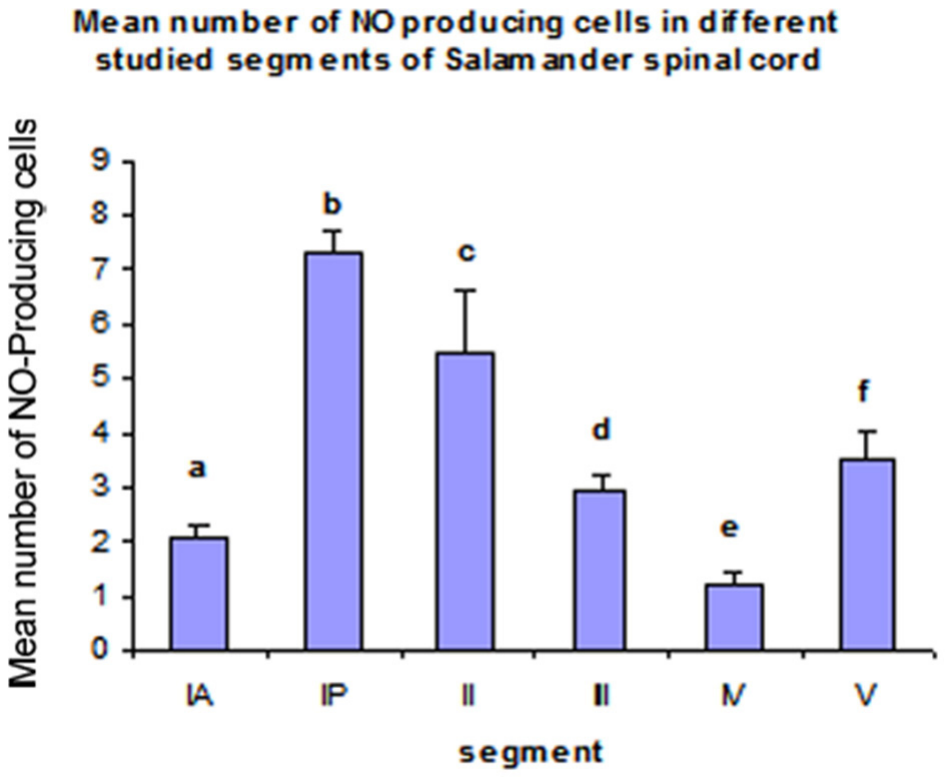
**Histograms illustrating the differences in the distribution of NO-PC along the spinal cord segments in salamanders**. ^a^*P* < 0.05 vs. segments IP, II, III and V; ^b^*P* < 0.05 vs. all other segments; ^c^*P* < 0.05 vs. all other segments; ^d^*P* < 0.05 vs. segments IP, IV and V; ^e^*P* < 0.05 vs. all other segments except IA; ^f^*P* < 0.05 vs. all other segments except III.

**Figure 8:**
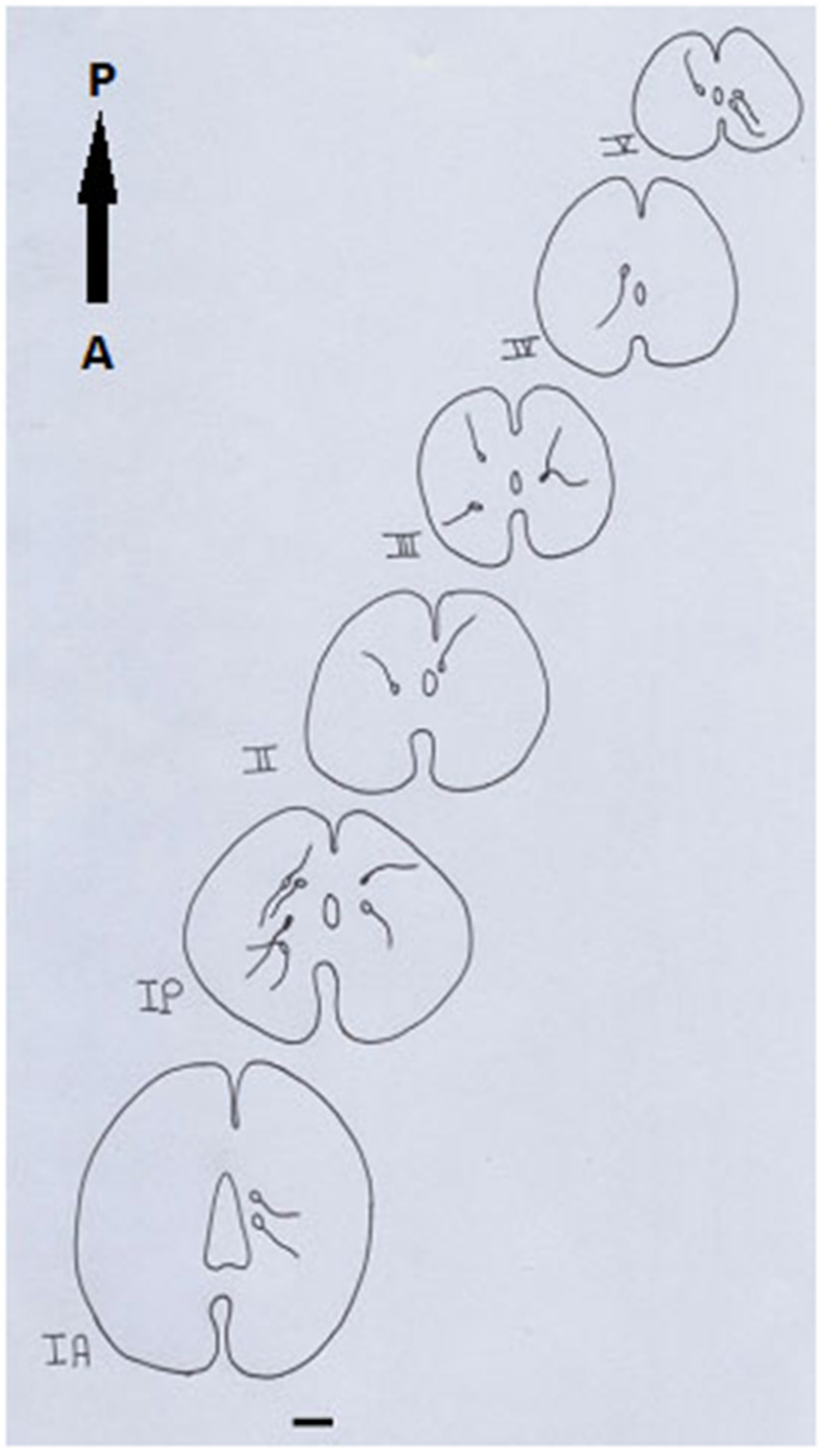
**Camera Lucida drawing shows the distribution of NADPH-d-positive neurons through the spinal cord length in salamander**. Scale bar: 100 μm.

**Figure 9:**
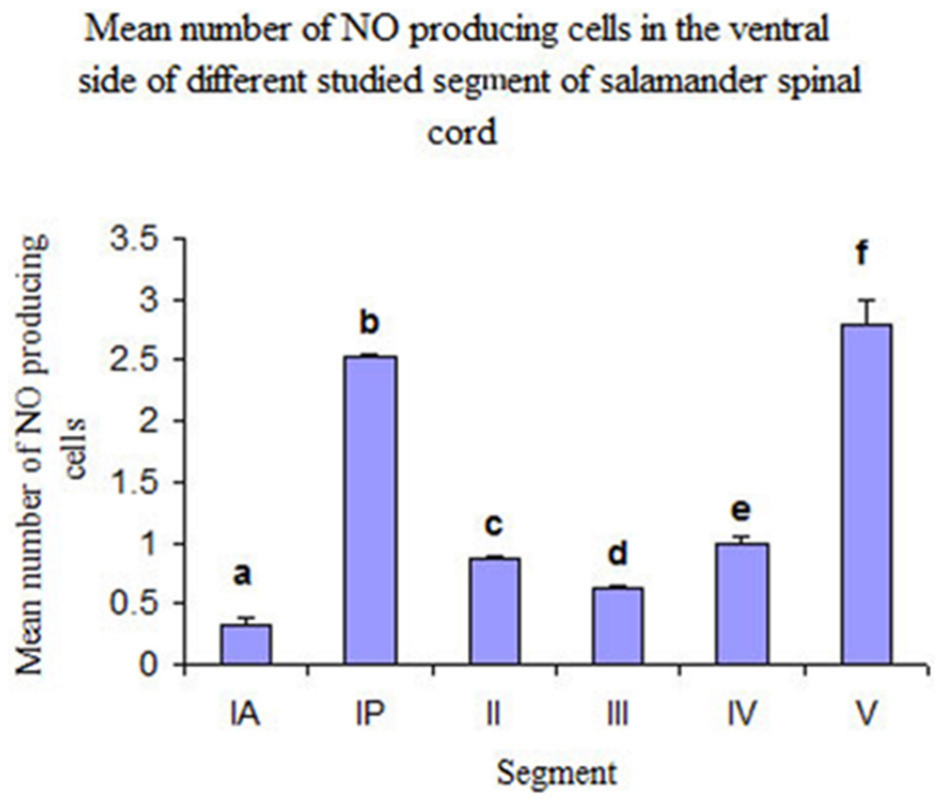
**Histograms illustrating the mean number of NO-PC in the ventral side of different spinal cord segments in salamander**. ^a^*P* < 0.05 vs. all other segments except III; ^b^*P* < 0.05 vs. all other segments except V; ^c^*P* < 0.05 vs. segments IA, IP and V; ^d^*P* < 0.05 vs. segments IP, V; ^e^*P* < 0.05 vs. segments IA, IP and V; ^f^*P* < 0.05 vs. all other segments except IP.

Mapping and distribution of labeled cells in representative transverse sections have been charted at different regions of the spinal cord as follows (Table 1):

Segment IA represented the anterior part of the spinal cord. Nitric oxide positive cells (NO-PC) was scarce and their number did not exceed 2 cells/section (Figure 3A). These cells were mainly found in the ventral side of the spinal cord and they were weakly labeled (arrows in Figure 3A). In segment IP, NO-PC significantly increased in number to reach the maximum cell number among all segments (7 cells/section) (Figure 3B). Some of these cells were highly stained compared to others; they were generally located in the periphery of the gray matter whereas long nerve fibers were ventrally directed (arrowhead in Figure 3B).
In segment II, NO-PC showed slight decrease in number (average 5 cells/section) compared to the previous one and almost all recognizable cells were highly labeled with NADPH-d (Figures 4A,B). In segment III, NO-PC showed more decline in number of positive cells (2 cells/section), that had different degree of labeling intensity. The cells with higher NADPH-d stain intensity were located in the ventral side of the spinal cord (Figure 5A). The axons of these cells had no specific direction. The least number of NO-PC was found in segment number IV (1 cell/section) (Figure 5B), where the intensity of NADPH-d labeling was faint and the cells axons were ventrally directed.
In segment V, the number of NO-PC significantly increased again (3 cells/section), in which they had higher NADPH-d staining intensity than in the previous segments (Figure 6). Most of these cells axons were also ventrally directed.
Comparing the distribution of NADPH-d positive neurons along spinal cord segments of pleurodeles, we found that the segment that had the most significant increase in number of NADPH-d positive cells was the IP segment (7.3 ± 0.38), then it gradually decreased posteriorly till it reached its minimum in segment IV (1.2 ± 0.2), then it started to increase again at the last segment (3.5 ± 0.57) (Figure 7).
A Camera Lucida drawing (Figure 8) showed NO-PC mapping through spinal cord segments. In segment IA, NO-PC appeared to have light staining and were located around the central canal. Their number was low compared to the following segment. In segment IP, more NO-PC were seen, some of which had dark and high NADPH-d stain ability. Their dendrites were mostly directed toward the ventral side, while other cells had faint staining. In segment II, the number of NO-PC was less than the previous one and all cells had a faint stain ability. Their dendrites were mostly directed toward the dorsal side. In segment III, some NO-PC got dark staining and others had lighter staining. Some of their dendrites were directed toward the dorsal side of the spinal cord while others were directed toward the ventral side. In segment IV, the number of NO-PC was less than any other segment of the spinal cord and cells had faint NADPH-d staining. Their dendrites were directed toward the ventral side of the cord. In the fifth segment, it was noticed that the number of NO-PC increased and their dendrites were mainly directed toward the ventral side.
In addition, it was noticed that NO-PC were accumulated in the ventral region of certain segments in the spinal cord, we counted the labeled cells in this region (Table 1). It was found that the segment that had the highest number of NO-PC was the IP segment (2.5 cells/section), then it gradually decreased along the rostrocaudal axis till segment number III, where it reached its minimum (0.6 cells/section) (Figure 9). Then, the NO-PC significantly increased again in segments IV and V reaching 2.8 cells/section.

**Table 1:** **Showing total number of NO-PC in different segments of salamander's spinal cord**.

**Segment**	**Mean number of cells/section**	**Mean number of cells/section in ventral side**
IA	2.03 ± 0.276	0.33 ± 0.05
IP	7.3 ± 0.38	2.53 ± 0.01
II	5.5 ± 1.08	0.86 ± 0.03
III	2.9 ± 0.28	0.63 ± 0.01
IV	1.2 ± 0.20	1.0 ± 0.06
V	3.5 ± 0.57	2.8 ± 0.2

*Mean* ± *SE*.

*p ≤ 0.05 vs. IA*.

## Discussion

The results of the present study provide a detailed map of the distribution of NADPH-d-containing neurons in the spinal cord of salamanders to localize NO, since the relative simplicity of the amphibian tadpole nervous system has been used as a model for the mechanisms underlying the generation and development of vertebrate locomotion (McLean et al., 2000).

From histochemistry experiments on salamanders, it was observed that the number of NO-PC per section was gradually decreasing toward the posterior end of the spinal cord; however, it suddenly increased just before the caudal region. Moreno et al. (2002) indicated that most, if not all nitrergic neurons in the spinal cord of amphibians are interneurons. Thus, we suggest that the NO-producing cells are interneurons especially in segments IP and V.

The highest immunoreactivity of NADPH-d was found in segment IP and this segment is at the forelimbs level. It was also found that NO-PC were mainly accumulated in the ventral region of the spinal cord, where it contains the somatic motoneurons of the spinal cord. The present data are in agreement with previous research (Crowe et al., 1995) where NADPH-d was present in neuronal populations within *Xenopus leavis* spinal cord, such as the fore and hind limb motoneuron pools. NO has also been reported to play a role in motoneuron development. Abundance of NADPH-d in certain segments of spinal cords, which are parallel to the fore and hind limb, implicates that NO has an important role as neuronal messenger in these regions of the spinal cord. Collectively, from previous and present findings we may conclude that NO-producing cells are the control keys for locomotion. Furthermore, we found that NO-PC were also mainly located in the intermediate gray area of spinal segments corresponding to the thoracic region. According to Crowe et al. (1995), this intermediate area corresponds to the location of autonomic preganglionic neuronal cell bodies. Consequently, we may conclude that the positive neurons in this area of the cord may have an autonomic function. In addition, the present results were in accordance with those of Dun et al. (1992), who stated that immunoreactivity for nNOS were present in neurons in regions of the rodent spinal cord that were associated primarily with autonomic and sensory functions. However, it is not excluded that NO-PC might be glial cells. A double labeling for astrocytic markers and NADPH-d would be necessary to clarify this possibility.

Fox (1992) stated that during metamorphosis the movement of anuran amphibians gradually switches from undulatory oscillation of the trunk and tail during tadpole swimming to limb-based propulsion in frog and toad, necessitating a complete remodeling of their locomotory system as limbs grow and the tail regresses. In this respect, results obtained herein could be discussed as the number of NO-producing cells is higher in the ventral side of the spinal cord segments corresponding to brachial and sacral plexuses. In our work, the number of NO-PC were increased in the spinal cord segments corresponding to brachial and sacral plexuses and also at the beginning of the tail and this could be due to the fact that salamanders are tetrapods capable of both swimming and walking (ten Donkelaar et al., 2001). Our finding is antagonistic with Ramanathan et al. (2006), who showed that during early stages of metamorphosis, nitrergic neurons were excluded from regions where spinal limb circuits were forming. As metamorphosis progressed, NOS expression became distributed along the length of the spinal cord together with an increase in the number and intensity of labeled cells and fibers.

The analysis of NADPH-d-expressing and/or NOS-immunoreactive neurons in the spinal cord of different animal species (Vizzard et al., 1994b, 1995) have shown a morphologically heterogeneous pattern of NADPH-d-expressing neuronal pools ranging from bipolar, poorly branched NADPH-d-exhibiting neurons in the superficial dorsal horn to highly differentiated neurons in the pericentral region (lamina X), deep dorsal horn (laminae IV–V), and dorsal gray commissure containing widely branching NADPH-d-exhibiting neurons (Vizzard et al., 1994a; Burnett et al., 1995; Maršala et al., 1998). In general, NADPH-d staining has been seen in neurons and fibers in the superficial dorsal horn, in neurons around the central canal at all levels of the spinal cord, in the dorsal commissure, in the sacral parasympathetic nucleus (SPN), and in the intermediolateral cell column (IML) of the thoracolumbar segments. In addition, large numbers of NADPH-d stained neurons were found in the superficial dorsal horn and around the central canal of rabbit spinal cord (Maršala et al., 2007). On the contrary, our finding was antagonistic to the previous two researches, NO-PC were seen mostly accumulated in the ventral parts of the spinal cord. The differences between these two findings could be due taxa differences.

The distribution of neuronal nitric oxide synthase-immunoreactive (nNOS-ir) system was described in man (Egberongbe et al., 1994) and rat's (Bredt et al., 1991) brain as well as in cat and mouse's spinal cord (Dun et al., 1993). Moreover, the system was described in more limited cerebral regions, as the hippocampus, the striatum, or the hypothalamus in the rat (Ng et al., 1999). NADPH-d-positive elements have been described within the brain and spinal cord of rat (Vincent and Kimura, 1992; Freire et al., 2004), the spinal cord of rabbit and mouse (Kluchova et al., 2000), and the mouse cerebellum (Brüning, 1993). Recently, Bombardi et al. (2013) suggested that NO may be involved in spinal sensory and visceral circuitries. Giraldez-Perez et al. (2008) found a wide distribution of double labeled cells co-localizing NADPH-d and NOS in brain and spinal cord of goldfish.

NADPH-d-labeled cells were consistently seen in the regions of the intermediate gray matter in the spinal cord of *Xenopus leavis* (Crowe et al., 1995). A similar study of Maršala et al. (1999) has shown that the segmental distribution of NADPH-d-expressing neurons in the rabbit is comparable to that described in the spinal cord of other species such as rat, mouse, cat, dog, squirrel, and monkey with certain differences consisting in an increasing number of NADPH-d-expressing neurons along the rostrocaudal axis of the spinal cord and accumulation of NADPH-d-positive somata in the lower lumbar and sacral segments. A considerable increase in the cell body size of large, multipolar, pericentrally located NADPH-d-expressing neurons was seen. McLean et al. (2000) found that NO appears to be located in three distinct clusters of neurons in the brain stem of *Xenopus* larva where they may function as a modulator, which is released with other conventional transmitters.

In conclusion, NADPH-d stain along the rostrocaudal spinal cord segments of salamanders has shown quantitative and qualitative heterogenous appearance of NO-PC. These findings raise the possibility that NO synthesized by bipolar neurons and large multipolar NADPH-d expressing neurons located in the ventral horn may be involved in the motor control. In addition, the present work revealed the accumulation of NO-PC at specific segments in the spinal cord that are related to fore- and hindlimb movement and stepping.

### Conflict of interest statement

The authors declare that the research was conducted in the absence of any commercial or financial relationships that could be construed as a potential conflict of interest.
